# Orthobiologics are not all the same: a mechanism-based scoping review of PRP, cytokine-rich serum, and extracellular vesicle–based strategies in musculoskeletal conditions

**DOI:** 10.3389/fpain.2026.1772644

**Published:** 2026-08-17

**Authors:** Luigi Di Lorenzo, Alfonso M. Forte, Bruno De Meo, Francesco Forte, Carmine D'Avanzo, Hassan Zmerly

**Affiliations:** 1 Rehabilitation Department, Mediterranean Neurological Institute Neuromed (IRCCS), Pozzilli, Italy; 2 Dipartimento Vita e Salute, Link Campus University, Rome, Italy; 3 Rehabilitation Department, Gruppo Forte Research Center, Salerno, Italy; 4 ASREM Azienda Sanitaria Molise, Rehabilitation, Isernia, Italy

**Keywords:** autologous conditioned serum, extracellular vesicles, orthobiologics, platelet-rich plasma, regenerative medicine

## Abstract

Autologous blood-derived orthobiologics, including platelet-rich plasma and cytokine-rich serum, are widely used in musculoskeletal medicine. Extracellular vesicle–based approaches are increasingly investigated as related biologically active strategies, although they may derive from multiple autologous or allogeneic cellular and tissue sources. However, these biologics differ significantly in composition and mechanism of action, often leading to confusion in terminology and inconsistent clinical application. This scoping review critically maps current evidence (2020–2025) from randomized trials, systematic reviews, and meta-analyses to clarify the biological heterogeneity among these products. Platelet-based PRP acts primarily via growth factor release, supporting anabolic and reparative processes. Leukocyte- and monocyte-containing PRP formulations, conversely, engage immune modulation and inflammatory signaling pathways. Acellular cytokine-rich sera (e.g., autologous conditioned serum) offer immunomodulatory effects through soluble mediators like IL-1Ra. EVs and exosomes represent a promising but still experimental approach, functioning as paracrine modulators with unclear clinical efficacy. While some formulations yield similar clinical outcomes, these cannot be interpreted as biologically equivalent. The only triple-blind randomized trial on exosomes in knee osteoarthritis confirmed safety but no superiority over placebo. A mechanism-informed, indication-driven selection is essential to optimize treatment efficacy, avoid inappropriate expectations, and advance the field responsibly. This review proposes a biologically grounded classification of orthobiologics and highlights the need for standardized reporting, accurate product characterization, and rigorous clinical validation. Clinicians and researchers are urged to abandon the “one-size-fits-all” approach and embrace biological specificity when selecting orthobiologic therapies for musculoskeletal conditions.

## Introduction

Orthobiologic and related biologically active strategies—platelet-based, leukocyte-modulated, acellular cytokine-driven, and extracellular vesicle–based approaches. Among these, platelet-rich plasma (PRP) has emerged as one of the most widely adopted—and arguably most misunderstood—interventions (1).

Despite its popularity, the term “PRP” is routinely used as a clinical catch-all, encompassing a broad range of biologically distinct preparations. Some are primarily platelet-based, designed to deliver growth factors that support tissue repair (2). Others contain significant leukocyte and monocyte fractions, shifting the mechanism of action toward immune modulation and inflammatory signaling (3). Yet both are often referred to simply as “PRP,” creating a dangerous illusion of equivalence.This semantic and biological ambiguity has serious consequences: it clouds scientific interpretation, reduces reproducibility across studies, and leads to therapeutic choices that may be mechanistically inappropriate (1–3). A PRP formulation intended to dampen inflammation may, in fact, exacerbate it—depending entirely on its cellular content. This problem is not merely academic; it affects real- world outcomes and patient care.Complicating the picture further, acellular products such as autologous conditioned serum (CYTOKINE-RICH SERUM) and cytokine-rich sera have entered clinical practice, offering anti-inflammatory effects through soluble mediators like IL-1Ra, rather than through cells or platelets (4, 5). Most recently, extracellular vesicles (EVs), including exosomes, have drawn attention as next-generation orthobiologics with potential paracrine and regenerative functions—yet are often conceptually lumped together with PRP despite operating through entirely different biological routes (6). In this fragmented and rapidly evolving landscape, clinicians and researchers are left to navigate a crowded field of orthobiologic options without a unified conceptual map. What is needed is a mechanism-based classification of blood-derived orthobiologics and related biologically active strategies—one that acknowledges their distinct compositions, biological actions, and clinical roles. The aim of this scoping review is to address that need. We present a structured, evidence-informed overview of the major categories of orthobiologic and related biologically active strategies—platelet-based, leukocyte-modulated, acellular cytokine-driven, andextracellular vesicle–mediated approaches—with a critical focus on one practical question: *who does what in musculoskeletal regenerative medicine?*

## Methods

A scoping review of the literature was conducted to map and critically synthesize the current evidence on orthobiologic and related biologically active strategies used in musculoskeletal and regenerative medicine. The review followed the methodological framework proposed by Arksey and O'Malley and was reported according to the PRISMA-ScR (Preferred Reporting Items for Systematic Reviews and Meta-Analyses extension for Scoping Reviews) guidelines.

A literature search was performed using PubMed, Scopus, and Web of Science databases. Studies published between January 2020 and October 2025 were screened. The search focused on platelet-rich plasma (PRP), leukocyte-rich PRP (LR-PRP), leukocyte-poor PRP (LP-PRP), Cytokine-Rich Serum (CRS)), cytokine-rich serum, extracellular vesicles (EVs), exosomes, and related orthobiologic biologically active products used in musculoskeletal conditions. The search strategy combined controlled vocabulary terms and free-text keywords including “platelet-rich plasma”, “PRP”, “leukocyte-rich PRP”, “leukocyte-poor PRP”, “autologous conditioned serum”, “cytokine-rich serum”, “extracellular vesicles”, “exosomes”, “orthobiologics”, “osteoarthritis”, “tendinopathy”, and “musculoskeletal disorders”. To improve comprehensiveness, reference lists of relevant articles and systematic reviews were also manually screened.

Eligible studies included randomized controlled trials (RCTs), systematic reviews, meta-analyses, high-quality scoping reviews, and registered clinical trials investigating orthobiologic or biologically active interventions in adult patients with musculoskeletal disorders. The interventions of interest included platelet-based preparations (PRP), leukocyte- and monocyte- containing blood-derived products, acellular cytokine-rich serum preparations such as Cytokine-Rich Serum (CRS)), and extracellular vesicle–based approaches including exosomes and small extracellular vesicles. Extracellular vesicles and exosomes were included as related biologically active orthobiologic strategies, although they are not necessarily autologous nor blood-derived, as they may originate from multiple cellular and tissue sources, including mesenchymal stromal cells and placental tissues.

Excluded studies included animal studies, purely *in vitro* or preclinical experimental studies, single case reports, non-English publications, studies unrelated to musculoskeletal medicine, and studies investigating exclusively pharmacological interventions without a biologically derived component.

The research question was structured according to the PICOS framework. The population included adult patients with musculoskeletal disorders. Interventions comprised PRP formulations, leukocyte-containing blood-derived products, cytokine-rich serum preparations, and EV/exosome-based approaches. Comparators, when applicable, included placebo, corticosteroids, standard care, or alternative orthobiologic formulations. Outcomes included pain relief, functional improvement, imaging findings, biomarker modulation, safety outcomes, and follow-up data.

Titles and abstracts were independently screened by investigators, followed by full-text eligibility assessment. Discrepancies were resolved through discussion and consensus with the senior author. Data were charted thematically according to biological composition and mechanism of action into four principal domains: platelet-driven growth factor–based approaches; leukocyte- and monocyte-mediated inflammatory/immunomodulatory approaches; acellular cytokine-mediated strategies; and extracellular vesicle–based translational approaches.

Consistent with the aims of a scoping review, this work was designed to provide a broad mapping of the available evidence rather than a quantitative pooled efficacy analysis. The review sought to identify areas of biological heterogeneity, methodological variability, evidence redundancy, and translational gaps within the orthobiologic field. This review did not have a prospectively registered protocol. Ethical approval and informed consent were not required because the study was based exclusively on previously published literature without patient involvement.

## Results

**Figure 1:**
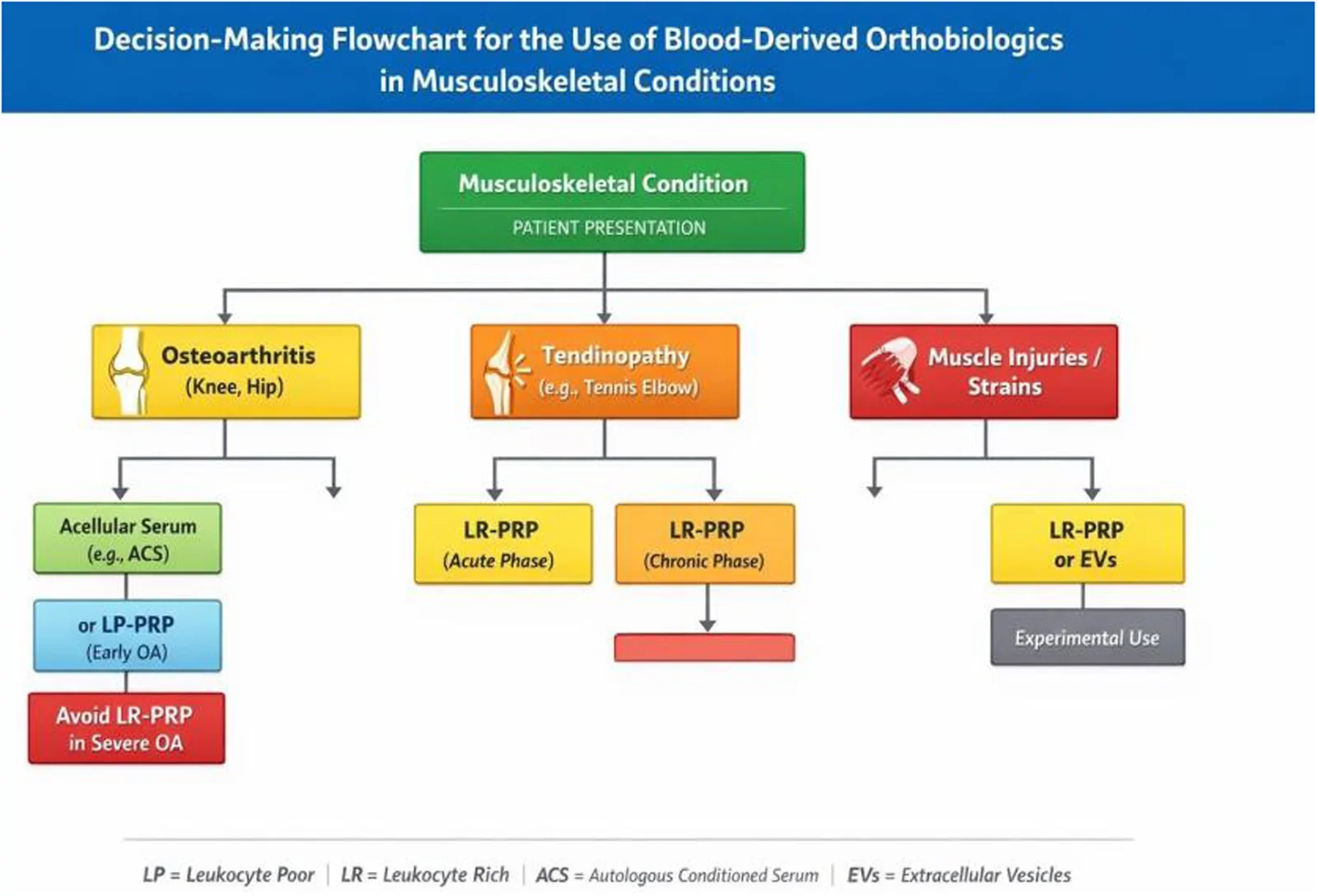
Decision-making flowchart for the selection of orthobiologic and related biologically active strategies. This algorithm synthesizes current evidence and biological rationale for product selection across osteoarthritis, tendinopathies, and muscle injuries. LP-PRP, leukocyte-poor platelet-rich plasma; LR-PRP, leukocyte-rich PRP; CYTOKINE-RICH SERUM, autologous conditioned serum; EVs, extracellular vesicles.

The study selection process is summarized in the PRISMA-ScR flow diagram (Figure 1).

Study characteristics, synthesized findings, and methodological quality assessment of the included studies are summarized in Tables 1–4.

**Table 1:** PRISMA-ScR flow diagram of study identification, screening, eligibility assessment, and inclusion for the scoping review of orthobiologic and related biologically active strategies in musculoskeletal medicine (2020–2025).

PRISMA-ScR phase	Description	Number of records
Identification	Records identified through PubMed	*n* = 24
Identification	Records identified through Scopus	*n* = 14
Identification	Records identified through Web of Science	*n* = 8
Identification	Additional records identified through manual reference screening and other sources	*n* = 2
Identification	Total records identified	*n* = 48
Identification	After manual verification no duplicate records were identified	*n* = 0
Screening	Records screened by title and abstract	*n* = 48
Screening	Records excluded after title/abstract screening	*n* = 27
Eligibility	Full-text articles assessed for eligibility	*n* = 21
Eligibility	Full-text articles excluded	*n* = 3
Eligibility	Exclusion reason: non-musculoskeletal focus	*n* = 1
Eligibility	Exclusion reason: preclinical or animal-only studies	*n* = 1
Eligibility	Exclusion reason: pharmacological/non-biologic interventions only	*n* = 1
Included	Studies included in the qualitative scoping synthesis	*n* = 18
Included	Included randomized controlled trials	*n* = 5
Included	Included systematic reviews/meta-analyses	*n* = 5
Included	Included high-quality scoping reviews/editorial evidence syntheses	*n* = 3
Included	Included registered ongoing clinical trials	*n* = 2
Included	Additional narrative/mechanistic background references	*n* = 3

**Table 2:** Characteristics and main findings of included studies on orthobiologic and related biologically active strategies in musculoskeletal medicine (2020–2025).

Study	Country	Study design	Population/setting	Product/intervention	Comparator	Outcomes	Follow-up	Main findings	Source/reference
Di Martino et al. (7)	Italy	Double-blind RCT	Knee osteoarthritis	LR-PRP intra- articular injections	LP-PRP	Clinical outcomes, safety	12 months	Both formulations improved symptoms; leukocyte content did not significantly influence efficacy or safety	Ref. (7)
Gupta et al. (8)	India	Editorial/Evidence synthesis	Knee osteoarthritis	Critical discussion of LR-PRP vs LP- PRP	—	Interpretation of biological heterogeneity	—	Similar outcomes should not be interpreted as biological equivalence	Ref. (9)
Vitali et al. (9)	Italy	Observational clinical study	Knee osteoarthritis	Cytokine-Rich Serum (, weekly injections	—	Pain and functional PROMs	Short-term follow-up	CYTOKINE-RICH SERUM associated with clinical improvement	Ref. (10)
Raeissadat et al. (10)	Iran	Systematic review and meta-analysis	Knee osteoarthritis	Cytokine-Rich Serum intra-articular injections	Various comparators	VAS, WOMAC	Variable	Cytokine-Rich Serum associated with improvement in pain and function	Ref. (11)
Damjanov et al. (11)	Serbia	Randomized double-blind controlled trial	Knee osteoarthritis	Triamcinolone + Cytokine-Rich Serum	Triamcinolone alone	Pain, function, QoL	24 weeks	Potential additive benefit of Cytokine-Rich Serum (CRS)	Ref. (12)
Ipek et al. (12)	Turkey	Pilot study	Lateral epicondylitis	Intratendinous Cytokine-Rich Serum	—	Pain and function	Short- and long-term	Promising preliminary findings	Ref. (13)
Curtis et al. (13)	USA	Observational study	Osteoarthritis	Cytokine-Rich Serum	—	PROMs	Variable	Improvement in patient- reported outcomes	Ref. (14)
Jeyaraman et al. (14)	India	Systematic review	Knee osteoarthritis	Cytokine-Rich Serum (CRS)	Various comparators	Clinical PROMs	Variable	Clinical interest confirmed, but methodological heterogeneity persists	Ref. (15)
Bolandnazar et al. (15)	Iran	Triple-blind placebo- controlled RCT	Bilateral knee osteoarthritis	Placental MSC- derived EVs	Saline placebo	VAS, WOMAC, Lequesne, MRI	6 months	Favorable safety profile but no superiority over placebo	Ref. (16)
NCT06937528	Multicenter	Registered clinical trial	Knee osteoarthritis	Intra-articular EVs	Dose comparison	Safety and feasibility	Ongoing	Early translational investigation	Ref. (17)
NCT06431152/NCT05060107	Multicenter	Registered clinical trial	Knee osteoarthritis	MSC-derived sEVs/exosomes	—	Safety and feasibility	Ongoing	Experimental translational pipeline	Ref. (18)

**Table 3:** Methodological quality assessment of included studies.

Study	Study design	Randomization/blinding	Control group	Validated clinical outcomes	Adequate follow-up	Methodological reporting	Overall methodological quality
Di Martino et al. (7)	Double-blind RCT	Yes/Double-blind	Yes (LR-PRP vs LP-PRP)	Yes	Yes (12 months)	Clear and detailed	High
Gupta et al. (8)	Editorial/Evidence synthesis	N/A	N/A	N/A	N/A	Transparent critical synthesis	Moderate
Vitali et al. (9)	Observational study	No	No	Yes	Not clearly specified	Descriptive	Moderate
Raeissadat et al. (10)	Systematic review and meta-analysis	Systematic process reported	N/A	Yes	Variable	Methodology declared	High
Damjanov et al. (11)	Randomized double-blind controlled trial	Yes/Double-blind	Yes	Yes	Yes (24 weeks)	Clear	High
Ipek et al. (12)	Pilot study	No	No	Yes	Limited	Adequate for pilot design	Low–Moderate
Curtis et al. (13)	Observational study	No	No	Yes	Not clearly specified	Descriptive	Moderate
Jeyaraman et al. (14)	Systematic review	Explicit criteria reported	N/A	Yes	Variable	Methodology declared	High
Bolandnazar et al. (15)	Triple-blind placebo- controlled RCT	Yes/Triple-blind	Yes (placebo)	Yes	Yes (6 months)	Highly detailed	High
NCT06937528	Registered clinical trial	Planned	Planned	Planned	Ongoing	Protocol registered	Not assessable
NCT06431152/NCT05060107	Registered clinical trial	Planned	Planned	Safety-focused	Ongoing	Protocol registered	Not assessable

**Table 4:** Evidence matrix of included orthobiologic and related biologically active strategies in musculoskeletal medicine.

Biological domain	Study	Study design	Population/setting	Intervention	Comparator	Reported outcomes	Level of evidence
Platelet-based PRP	Di Martino et al. (7)	Double-blind RCT	Knee osteoarthritis	LR-PRP	LP-PRP	Clinical outcomes and safety	High

The literature analysis confirmed a marked biological and methodological heterogeneity among autologous blood-derived orthobiologic products, despite their frequent aggregation under overlapping terminology. Based on composition, mechanism of action, and clinical intent, the retrieved evidence could be mapped into four principal domains: platelet-based PRP preparations, leukocyte- and monocyte-containing blood-derived products, acellular cytokine-rich sera, and extracellular vesicles/exosomes. The characteristics and outcomes of the included studies are summarized in Tables 2–5.

**Table 5:** Standardized classification of orthobiologic and related biologically active strategies.

Product category	Biological composition	Primary mechanism of action	Main clinical indications	Level of clinical evidence
Leukocyte-Poor PRP (LP-PRP)	Platelets suspended in plasma with minimal or no leukocytes	Platelet-derived growth factor release; anabolic and reparative signaling	Mild-to-moderate osteoarthritis; chronic tendinopathies with low inflammatory activity	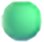 **High**
Leukocyte-Rich PRP (LR-PRP)	Platelets plus leukocytes and monocytes	Immune cell-mediated inflammatory and immunomodulatory activity	Acute or subacute tendinopathies; muscle injuries where controlled inflammation may be beneficial	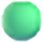 **Moderate**
Cytokine-Rich Serum (CRS)	Cell-free serum enriched with soluble cytokines (e.g., IL-1Ra)	Anti-inflammatory and immunomodulatory effects via soluble mediators	Knee osteoarthritis; adjunctive therapy to intra-articular corticosteroids; patients with an inflammatory phenotype	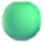 **High**
Extracellular Vesicles/Exosomes (EVs)	Nano-sized vesicles containing proteins, lipids, and RNA	Paracrine intercellular signaling and modulation of cellular behavior	Experimental use only; early clinical research mainly in knee osteoarthritis	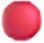 **Low (Experimental)**

CRS (Cytokine-Rich Serum) includes autologous conditioned serum and related acellular cytokine-enriched serum preparations.

Platelet-Based and Leukocyte-Containing PRP Preparations. The strongest comparative evidence concerned leukocyte-rich (LR-PRP) and leukocyte-poor PRP (LP-PRP) formulations in knee osteoarthritis. Di Martino et al. reported significant clinical improvement in both treatment groups, without significant differences in efficacy, adverse events, or failure rates during 12 months of follow-up. These findings suggest that both formulations may provide clinical benefit, although their biological composition differs substantially (7), adverse events, and failure rates between the two formulations across multiple follow-up time points. Both studies converged on the finding that leukocyte content, within the tested protocols, did not significantly influence overall clinical efficacy or safety. These findings were further contextualized by an editorial evidence synthesis by Gupta (8), which critically discussed the interpretation of LR-PRP vs. LP-PRP trials. The author emphasized that comparable outcomes between formulations should not be interpreted as biological equivalence, highlighting the need for precise product characterization and cautioning against indiscriminate use of the PRP label without compositional transparency.

Acellular Cytokine-Rich Serum Preparations. A distinct body of evidence addressed acellular cytokine-rich sera, particularly autologous conditioned serum, predominantly in knee osteoarthritis but also in selected tendinopathies. Unlike PRP-based approaches, these preparations are characterized by the absence of cellular components and rely on soluble mediators for their biological effects.

Early clinical evidence within the selected time frame includes the study by Vitali et al. (9), which evaluated cytokine-rich serum administered as four weekly intra-articular injections in patients with knee osteoarthritis. The study focused on changes in pain and functional patient-reported outcomes following the treatment cycle. A broader synthesis of randomized evidence was provided by the meta-analysis by Raeissadat et al. (10), which reported improvements in pain and function, as measured by VAS and WOMAC scores, across included trials, although with heterogeneity in protocols and follow-up duration.

The role of Cytokine as an adjunctive therapy was explored in a controlled double-blind randomized study by Damjanov et al. (11), in which Cytokine-Rich Serum was administered in combination with intra-articular triamcinolone. Outcomes related to pain, function, and quality of life were assessed up to 24 weeks, providing evidence on the potential additive role of cytokine-rich serum in an established pharmacological framework. Beyond osteoarthritis, Ipek et al. (12) investigated intratendinous CYTOKINE-RICH SERUM injections in patients with lateral epicondylitis in a pilot study, reporting short- and long-term clinical outcomes and identifying the approach as promising while underscoring the need for larger trials. More recent observational evidence by Curtis et al. (13) focused on patient-reported outcome measures in osteoarthritis following Cytokine-Rich Serum (CRS) treatment, while a systematic review by Jeyaraman et al. (14) synthesized available clinical studies on Cytokine-Rich Serum (CRS) in knee osteoarthritis, confirming clinical interest but also methodological variability.

Extracellular Vesicles and Exosomes. Evidence regarding extracellular vesicles (EVs) and exosomes in musculoskeletal disorders was substantially more limited and predominantly exploratory. Only one high-quality randomized controlled clinical trial was identified within the selected time frame. In a triple-blind, placebo-controlled randomized trial, Bolandnazar et al. (15) evaluated intra-articular injection of extracellular vesicles derived from placental mesenchymal stromal cells in patients with bilateral knee osteoarthritis, using a within-patient randomization design. Outcomes included pain and function scores (VAS, WOMAC, Lequesne) as well as MRI assessment at six months. The study demonstrated a favorable safety profile but did not show superiority over placebo for either clinical or imaging outcomes. In addition to completed trials, several registered clinical trials were identified, including NCT06937528 and NCT06431152/NCT05060107 (16, 17), investigating intra-articular administration of EVs or small extracellular vesicles (sEVs) in knee osteoarthritis, primarily focusing on safety, feasibility, and dose exploration. These ongoing studies highlight an active translational pipeline but do not yet provide definitive evidence of clinical efficacy.

## Discussion

This scoping review confirms that orthobiologic and related biologically active strategies used in musculoskeletal medicine should not be considered a single therapeutic category, but rather a spectrum of biologically distinct interventions characterized by different compositions, mechanisms of action, and levels of clinical maturity. The frequent use of umbrella terminology, particularly the generalized use of the term “PRP”, obscures substantial biological heterogeneity and contributes to inconsistent interpretation of clinical outcomes across studies.

The evidence reviewed in this study highlights that platelet-based preparations, leukocyte- and monocyte-containing blood-derived products, acellular cytokine-rich serum formulations, and extracellular vesicle–based approaches differ substantially in both biological rationale and mechanism of action. Platelet-rich plasma formulations primarily act through platelet-derived growth factor release and tissue repair signaling, whereas leukocyte-containing preparations may additionally engage inflammatory and immunomodulatory pathways through immune-cell activity. These differences are often insufficiently reported in clinical studies, limiting reproducibility and meaningful comparison between trials.

Within platelet-based interventions, the strongest comparative clinical evidence currently derives from randomized controlled trials evaluating leukocyte-rich and leukocyte-poor PRP in knee osteoarthritis. The reviewed studies generally reported comparable clinical outcomes and safety profiles between formulations within specific treatment protocols. However, similar clinical outcomes should not be interpreted as evidence of biological equivalence. Rather, these findings support the concept that distinct biological mechanisms may lead to partially overlapping clinical effects depending on disease phenotype, inflammatory environment, timing of intervention, and outcome measures used.

Acellular cytokine-rich serum preparations, including autologous conditioned serum, represent a conceptually different strategy from platelet-based interventions. These products are characterized by the absence of cellular components and rely predominantly on soluble mediators with anti-inflammatory and immunomodulatory activity. The available evidence suggests potential benefits in osteoarthritis and selected tendinopathies, although heterogeneity in preparation protocols, treatment schedules, and outcome reporting remains substantial. Current evidence supports considering cytokine-rich serum preparations as a separate biologically active category rather than a variation of PRP.

Extracellular vesicles and exosomes occupy a distinct and currently translational domain within the orthobiologic landscape. Importantly, EV-based products should not automatically be categorized as autologous blood-derived interventions, as they may originate from several cellular and tissue sources, including mesenchymal stromal cells, placental tissues, adipose-derived cells, and platelet-derived products. The available clinical evidence in musculoskeletal medicine remains limited. The only identified triple-blind randomized placebo- controlled trial in knee osteoarthritis evaluated placental mesenchymal stromal cell-derived extracellular vesicles and demonstrated an acceptable safety profile but no superiority over placebo on clinical or imaging outcomes at six months. Ongoing registered trials indicate sustained scientific interest; however, current evidence remains preliminary and largely translational.

Collectively, these findings suggest that terminological oversimplification and inadequate biological characterization remain major barriers to evidence synthesis in orthobiologic research. Grouping mechanistically different products under shared terminology may contribute to inconsistent literature, unrealistic therapeutic expectations, and difficulties in comparing outcomes across studies. Accurate reporting of product composition, leukocyte content, preparation methods, and biological rationale therefore appears essential for both scientific rigor and responsible clinical application.

From a methodological perspective, this scoping review was designed to provide a broad mapping of current evidence rather than a quantitative pooled efficacy analysis. The inclusion of randomized controlled trials, systematic reviews, meta-analyses, and registered translational studies allowed identification of evidence clusters, areas of biological overlap, and important gaps requiring further investigation. The findings support the need for future mechanism-informed clinical trials with standardized reporting frameworks and clearer biological characterization of orthobiologic products.

This review has several limitations. First, it was not prospectively registered. Second, only English-language studies were included. Third, the scoping methodology was designed to map evidence rather than perform a quantitative efficacy synthesis. Finally, heterogeneity of orthobiologic preparations limited direct comparison across studies.

## Conclusion

This scoping review demonstrates that orthobiologic and related biologically active strategies used in musculoskeletal medicine represent a heterogeneous and mechanistically diverse group of interventions rather than a unified therapeutic modality. Platelet-based PRP formulations, leukocyte- and monocyte-containing blood-derived products, acellular cytokine- rich serum preparations, and extracellular vesicle–based approaches differ substantially in biological composition, mechanism of action, and level of clinical validation and therefore should not be considered interchangeable despite their frequent overlap in terminology.

The currently available evidence suggests that comparable clinical outcomes across certain formulations, particularly between leukocyte-rich and leukocyte-poor PRP in knee osteoarthritis, do not necessarily imply biological equivalence. Similarly, cytokine-rich serum preparations and extracellular vesicle–based strategies operate through conceptually distinct pathways and should be interpreted within their specific biological context.

Accurate biological characterization, transparent reporting of preparation methods, and appropriate use of terminology are essential to improve reproducibility, facilitate meaningful comparison across studies, and support responsible clinical application. Extracellular vesicle–based therapies currently remain investigational in musculoskeletal medicine, with limited high-quality clinical evidence supporting their efficacy.

Future research should prioritize mechanism-informed study design, standardized reporting frameworks, clearer characterization of orthobiologic products, and adequately powered randomized controlled trials capable of distinguishing biological effects across different therapeutic strategies.

## Clinical interpretation points

Avoid LR-PRP in advanced osteoarthritis (Kellgren–Lawrence grade 3–4) or in joints with active synovitis, due to the risk of exacerbating inflammation through leukocyte-driven cytokine release.

- Prefer CYTOKINE-RICH SERUM or LP-PRP in cases with chronic low-grade inflammation or poor tolerance/response to NSAIDs. These options provide anti-inflammatory effects without high leukocyte content.
- Consider LR-PRP or EVs in acute soft tissue injuries, such as muscle strains or early tendinopathies, where controlled inflammation may facilitate tissue remodeling.
- Do not assume biological equivalence between LP-PRP and LR-PRP. Although clinical outcomes may appear similar in certain trials, their underlying cellular mechanisms and inflammatory profiles are fundamentally different.

## Data Availability

The original contributions presented in the study are included in the article/Supplementary Material, further inquiries can be directed to the corresponding author.
